# Increased plasma levels of galectin-1 in pancreatic cancer: potential use as biomarker

**DOI:** 10.18632/oncotarget.26034

**Published:** 2018-08-31

**Authors:** Neus Martinez-Bosch, Luis E. Barranco, Carlos A. Orozco, Mireia Moreno, Laura Visa, Mar Iglesias, Lucy Oldfield, John P. Neoptolemos, William Greenhalf, Julie Earl, Alfredo Carrato, Eithne Costello, Pilar Navarro

**Affiliations:** ^1^ Cancer Research Program, IMIM, Hospital del Mar Medical Research Institute, Unidad Asociade CSIC, Barcelona, Spain; ^2^ Department of Gastroenterology, Universidad Autonoma de Barcelona, Hospital del Mar, Barcelona, Spain; ^3^ Department of Medical Oncology, Hospital del Mar, Barcelona, Spain; ^4^ Department of Pathology, Universidad Autonoma de Barcelona, Hospital del Mar, CIBERONC, Barcelona, Spain; ^5^ Department of Molecular and Clinical Cancer Medicine, Institute of Translational Medicine, University of Liverpool, Liverpool, UK; ^6^ Department of General Surgery, University of Heidelberg, Heidelberg, Germany; ^7^ Department of Medical Oncology, Ramon y Cajal University Hospital, CIBERONC, IRYCIS, Alcala University, Madrid, Spain; ^8^ Institute of Biomedical Research of Barcelona (IIBB-CSIC), Barcelona, Spain

**Keywords:** galectin-1, pancreatic cancer, chronic pancreatitis, biomarker

## Abstract

Pancreatic ductal adenocarcinoma (PDA) is the most frequent type of pancreatic cancer and one of the deadliest diseases overall. New biomarkers are urgently needed to allow early diagnosis, one of the only factors that currently improves prognosis. Here we analyzed whether the detection of circulating galectin-1 (Gal-1), a soluble carbohydrate-binding protein overexpressed in PDA tissue samples, can be used as a biomarker for PDA. Gal-1 levels were determined by ELISA in plasma from healthy controls and patients diagnosed with PDA, using three independent cohorts. Patients with chronic pancreatitis (CP) were also included in the study to analyze the potential of Gal-1 to discriminate between cancer and inflammatory process. Plasma Gal-1 levels were significantly increased in patients with PDA as compared to controls in all three cohorts. Gal-1 sensitivity and specificity values were similar to that of the CA19-9 biomarker (the only FDA-approved blood test biomarker for PDA), and the combination of Gal-1 and CA19-9 significantly improved their individual discriminatory powers. Moreover, high levels of Gal-1 were associated with lower survival in patients with non-resected tumors. Collectively, our data indicate a strong potential of using circulating Gal-1 levels as a biomarker for detection and prognostics of patients with PDA.

## INTRODUCTION

Pancreatic ductal adenocarcinoma (PDA) is the most frequent type of pancreatic cancer and presents the worst prognosis of all tumors. It is currently the fourth leading cause of cancer-related deaths in Western countries and is predicted to rise to the second by 2030 [1]. The most accepted model for PDA progression is that tumors arise through the progressive accumulation of genetic alterations in normal cells, starting with non-invasive precursor lesions called pancreatic intraepithelial neoplasia (PanINs) and ending with infiltrating ductal adenocarcinoma [2]. K-Ras activation and telomere shortening are early molecular events in this pathway, while inactivation of INK4A/p16 and inactivating mutations of TP53 and SMAD4 occurs in intermediate (PanIN-2) or late stages of progression (PanIN-3), respectively [2]. Although PanINs are the most widely studied and common PDA precursors, extensive data indicate that intraductal papillary mucinous neoplasms (IPMNs) and mucinous cystic neoplasms (MCNs) are also important pancreatic preneoplastic lesions [3]. Risk factors associated with PDA etiology include smoking, obesity and chronic pancreatitis (CP) [4, 5]. CP is a severe disorder with an annual incidence ranging from 5–12/100,000 persons, and it leads to a significant reduction of quality of life [6]. Pancreatic inflammation accelerates PDA initiation and progression in mouse models of this disease [7]. As both PDA and CP initially display similar vague symptoms, such as abdominal pain, digestive symptoms, weight loss and inflammation, differential diagnosis between both diseases is very difficult at early stages—yet early diagnosis is crucial for treatment of PDA.

There are currently no effective or specific diagnostic methods to detect PDA at early stages. The most commonly used tests for diagnosis of pancreatic disorders are conventional imaging techniques [e.g., computed tomography scans], endoscopic ultrasound-guided fine-needle aspiration cytology and blood biomarkers. Among the different blood biomarkers for PDA, only CA19-9 (a sialylated Lewis^a^ antigen present in glycosphingolipids and glycoproteins) is approved by the US Food and Drug Administration (FDA) for clinical use. However, CA19-9 has important limitations, such as giving false negatives in patients with Lewis blood type negative phenotypes (Le^a–b–^) and false positives in patients with obstructive jaundice [8]. Additionally, CA19-9 is elevated in other tumors, some benign diseases and non-malignant inflammatory pathologies (including pancreatitis) [9]. CA19-9 is therefore not very useful for PDA diagnosis; in fact, its use by the clinicians is mostly restricted to checking for response to treatment or cancer recurrence [10]. Thus, identifying other biomarkers for early PDA detection is urgently needed.

Galectins are a family of 15 proteins that bind β-galactose–containing glycoconjugates through a highly conserved carbohydrate recognition domain (CRD). They can bind O- or N-linked glycans containing the basic core disaccharide N-acetyllactosamine (LacNAc), but each member displays glycan-binding specificities, leading to different biological functions [11]. Galectin-1 (Gal-1), the first identified member of the family, is a 14 kDa protein that can be located in the cell cytoplasm, nucleus, cell membrane and extracellular matrix. Gal-1 has a single CRD that recognizes preferentially non-sialylated and α2,3-sialylated complex N-glycans containing poly-N-LacNAc residues, although other variables, such as conformational changes of glycan motifs and protein-protein interactions, may account for its binding specificity [12]. Gal-1 has a wide range of biological functions, which are dictated by its concentration, cellular location and redox status [12]. Extracellular Gal-1 requires homodimerization (via its hydrophobic core) for functional activity; through its CRD, the homodimer can interact with glycosylated proteins to modulate cell adhesion, aggregation and migration [12–14]. In contrast, intracellular Gal-1 functions mainly as a monomer and, can trigger cell transformation via H-Ras protein-protein interactions [15] and modulate cellular functions, such as splicing [16]. Remarkably, one major role of Gal-1 is regulation of inflammation and the innate and adaptive immune responses, leading to its immunosuppressive effects [17, 18].

In cancer, Gal-1 is overexpressed in several tumors [19], including pancreatic cancer [20–23]. We have previously reported that this protein plays a pivotal role in PDA cancer progression by promoting tumor growth, angiogenesis, stroma activation and immune evasion [24, 25]. Moreover, Gal-1 has been suggested to be involved in resistance to cancer therapies [26–28]. Gal-1 is a small soluble molecule that can be secreted into the extracellular space through a non-canonical secretory pathway [29]. Accordingly, in addition to the overexpression of Gal-1 in tumor tissues, increased levels of this protein have been reported in plasma or serum from patients with different cancer types [30–35]. However, blood levels of Gal-1 in pancreatic cancer patients have not yet been analyzed.

In this study, we aimed to determine whether detection of Gal-1 circulating levels can be used as a clinical marker for PDA diagnosis and/or progression. We first analyzed the expression levels of Gal-1 by immunohistochemistry (IHC) in tissue samples from normal pancreas, CPs, preneoplastic lesions (PanINs, IMPNs) and PDA. Gal-1 plasma concentrations were analyzed via ELISA, using blood samples collected from a total 90 patients with PDA, 52 patients with CP and 28 healthy controls from three different cohorts. We also compared Gal-1 and CA19-9 levels as diagnostic markers for PDA and/or CP, either individually or in combination. Finally, we evaluated whether Gal-1 plasma levels correlate with PDA tumor stage, grade, metastasis and/or disease outcome. Our data show that circulating levels of Gal-1 are increased in pancreatic cancer, suggesting its usefulness as a novel biomarker for diagnosis and eventually prognosis of this fatal disease.

## RESULTS

### Expression of Gal-1 in normal, inflamed, preneoplastic and neoplastic human pancreatic tissues

**Figure 1:**
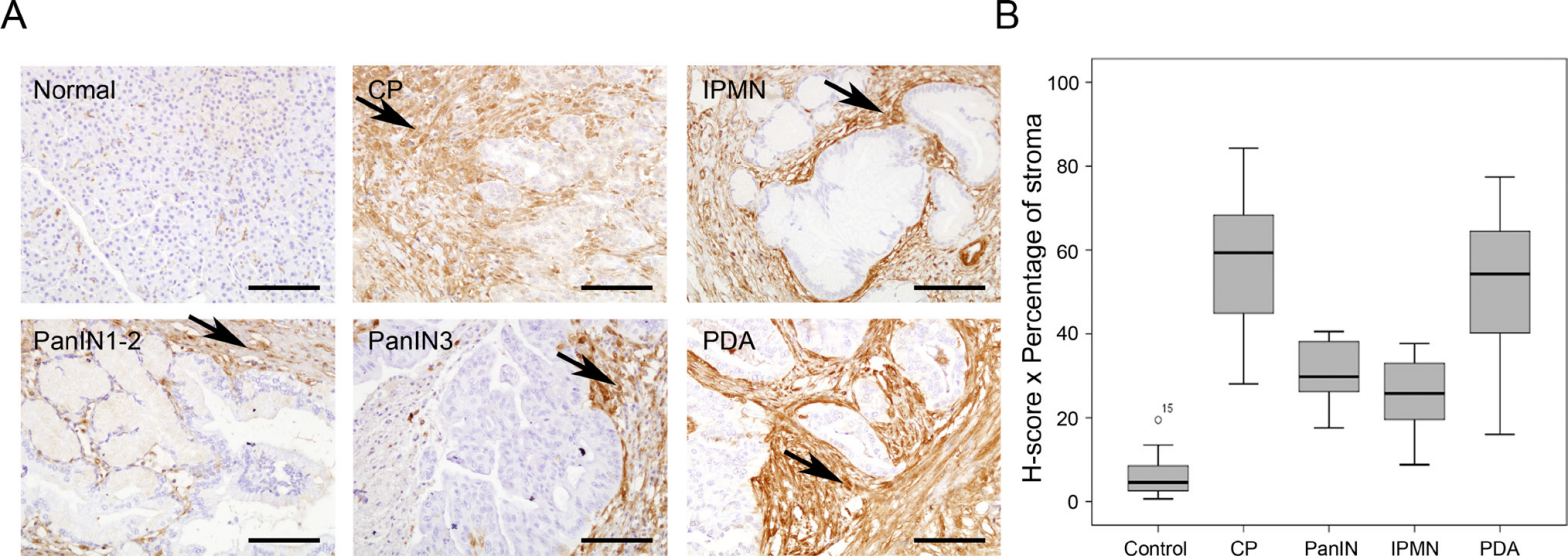
Gal-1 immunohistological expression in normal and pathological human pancreatic tissue samples (**A**) Immunostaining of Gal-1 in normal pancreas, CP, IPMN, low and high PanIN lesions and PDA. (**B**) Box-and-whisker plots showing H-scores corrected by the percentage of stroma in the tissue for normal pancreas, CP, PanINs, IPMN and PDA. Scale bars, 200 μm.

High Gal-1 tissue levels have been reported in pancreatic cancer in human [20, 22, 36] and mouse [23, 24]; however, its expression in CP and during different steps of PDA progression have not been thoroughly investigated. We thus analyzed Gal-1 expression by IHC in normal and pathological tissue pancreatic samples, including CP, different preneoplastic lesions (IPMN or PanIN) and PDA. Gal-1 was highly expressed by pancreatic stellate cells/fibroblasts associated with desmoplasia in CP, IPMN, low- or high-grade PanINs and PDA, but it was not detected in ductal cells in any tissue samples (Figure 1A and Supplementary Table 1). In the fibrotic stroma, Gal-1 was expressed in the cytoplasm and/or nuclei of pancreatic stellate cells and in the extracellular matrix. Quantification of Gal-1 protein expression levels by H-score showed similar intensity levels in all pathological samples; however, after H-scores were normalized with the percentage of stroma in each lesion type, Gal-1 expression was significantly higher in CP and PDA samples (Figure 1B and Supplementary Table 1). Thus, Gal-1 expression increased in pancreas during pathological conditions, with high levels in PDA and CP, due to the strong desmoplastic reaction present in these pathological conditions.

### Detection of Gal-1 in plasma from healthy controls compared to patients with CP or PDA

As Gal-1 can be secreted [29], we next analyzed circulating Gal-1 in healthy individuals and patients with CP or PDA. Three independent cohorts of patients were used: Barcelona-HM (*n* = 61 individuals), Liverpool-UL (*n* = 69 individuals) and Madrid-HURC (*n* = 40 individuals) (see Materials and Methods for breakdown of each cohort, and Supplementary Table 2 for clinicopathological data of patients). To avoid deviations due to sample handling and processing, each cohort was analyzed separately.

**Figure 2:**
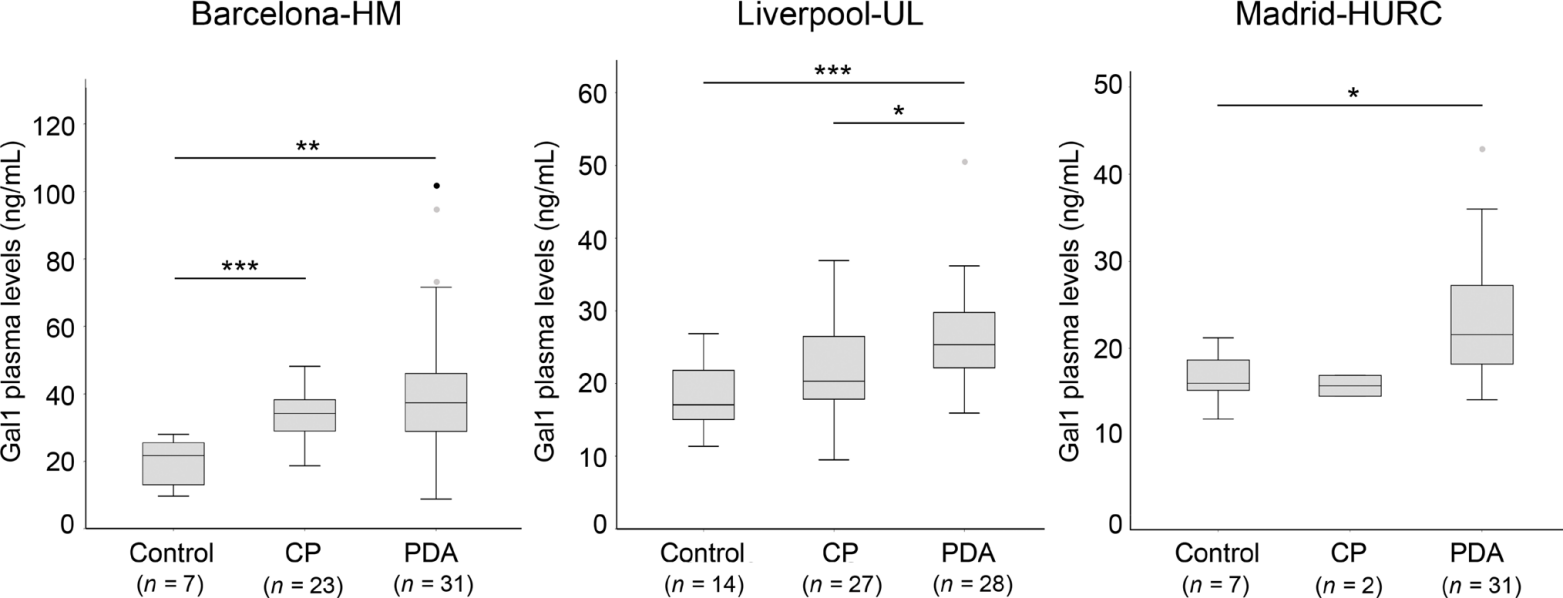
Plasma levels of Gal-1 from healthy controls, CP and PDA samples from the three different cohorts Box-and-whisker plot representation of Gal-1 levels in Barcelona-HM (left), Liverpool-UL (center) and Madrid-HURC (right) cohorts. ^*^p < 0.05; ^**^p < 0.01; ^***^p < 0.001 (Mann–Whitney test).

Notably, the median value of plasma Gal-1 levels (measured by ELISA) significantly increased for PDA patients as compared to healthy controls in all three cohorts (Barcelona-HM, 37.34 ng/ml compared to 21.62 ng/ml; Liverpool-UL, 25.36 ng/ml compared to 17.10 ng/ml; and Madrid-HURC, 21.6 ng/ml compared to 16.00 ng/ml, for PDA patients and controls, respectively) (Figure 2 and Table 1). We also found that Gal-1 levels increased in patients with CP as compared to controls (Barcelona-HM, 34.17 ng/ml compared to 21.62 ng/ml; Liverpool-UL, 20.34 ng/ml compared to 17.10 ng/ml), although significance was only reached for Barcelona-HM (note that the Madrid-HURC cohort comprised only 2 patients with CP and was not considered for statistical analysis) (Figure 2 and Table 1). Moreover, we detected significantly decreased levels of Gal-1 in patients with CP (20.34 ng/ml) as compared to those with PDA (25.36 ng/ml) for Liverpool-UL (Figure 2 and Table 1). Neither age nor sex was associated with circulating Gal-1 levels in healthy controls (Supplementary Tables 3 and 4).

**Table 1:** Gal-1 plasma levels detected by ELISA in the three independent cohorts

		Barcelona-HM				Liverpool-UL				Madrid-HUMC			
		*n*	Median	IQR	*p*	*n*	Median	IQR	*p*	*n*	Median	IQR	*p*
**Pathology**													
	**Ctl**	7	21.62	15.47		14	17.10	7.43		7	16	5.30	
	**CP**	23	34.17	9.58	<0.001	27	20.34	8.69	ns	2	15.70	-	ns
	**PDA**	31	37.34	18.71	0.001/0.396^*^	28	25.36	8.38	<0.001/0.031^*^	31	21.60	9.50	0.019/ns^*^
**TNM**													
	**I**	2	35.97	-		2	24.57	-					
	**II**	10	38.14	33.66	ns	18	25.36	8.90	ns	3	22.80	-	
	**III**	5	37.87	44.05	ns	0				6	19.95	14.55	ns
	**IV**	14	36.07	24.21	ns	8	26.24	9.22	ns	20	21.50	9.55	ns
	**N/A**									2	-	-	
**Grade**													
	**Low**	10	30.86	37.78		5	26.17	7.49		2	28.50	-	
	**High**	3	34.79	49.51	ns	18	23.84	10.36	ns	9	19.70	9.45	ns
	**N/A**	18	-	-		5	-	-		20	-	-	
**Metastasis**													
	**No**	17	36.30	17.04		20	25.20	7.16		10	20.65	9.77	
	**Yes**	14	37.30	26.28	ns	8	26.24	9.22	ns	21	21.60	10.70	ns

IQR, interquartile range; ns, not significant; ^*^p value of CP compared to PDA; N/A, not ascertained; TNM, tumor–node–metastasis cancer staging system.

High bilirubin concentrations are frequently found in serum/plasma of PDA patients (normally due to obstruction of the common bile duct during tumor growth) and can interfere with different assays [37]. However, we observed no significant differences in plasma Gal-1 levels in PDA patients with normal or high blood bilirubin levels (Supplementary Table 5). We also tested plasma Gal-1 levels in the context of diabetes, as this condition is frequently associated with PDA [38, 39], and Gal-1 levels are increased in type 2 diabetes [40]. However, we found no significant differences in plasma Gal-1 expression levels between non-diabetic and diabetic patients (Supplementary Table 5).

### Determination of plasma Gal-1 cut-off values for PDA detection

**Figure 3:**
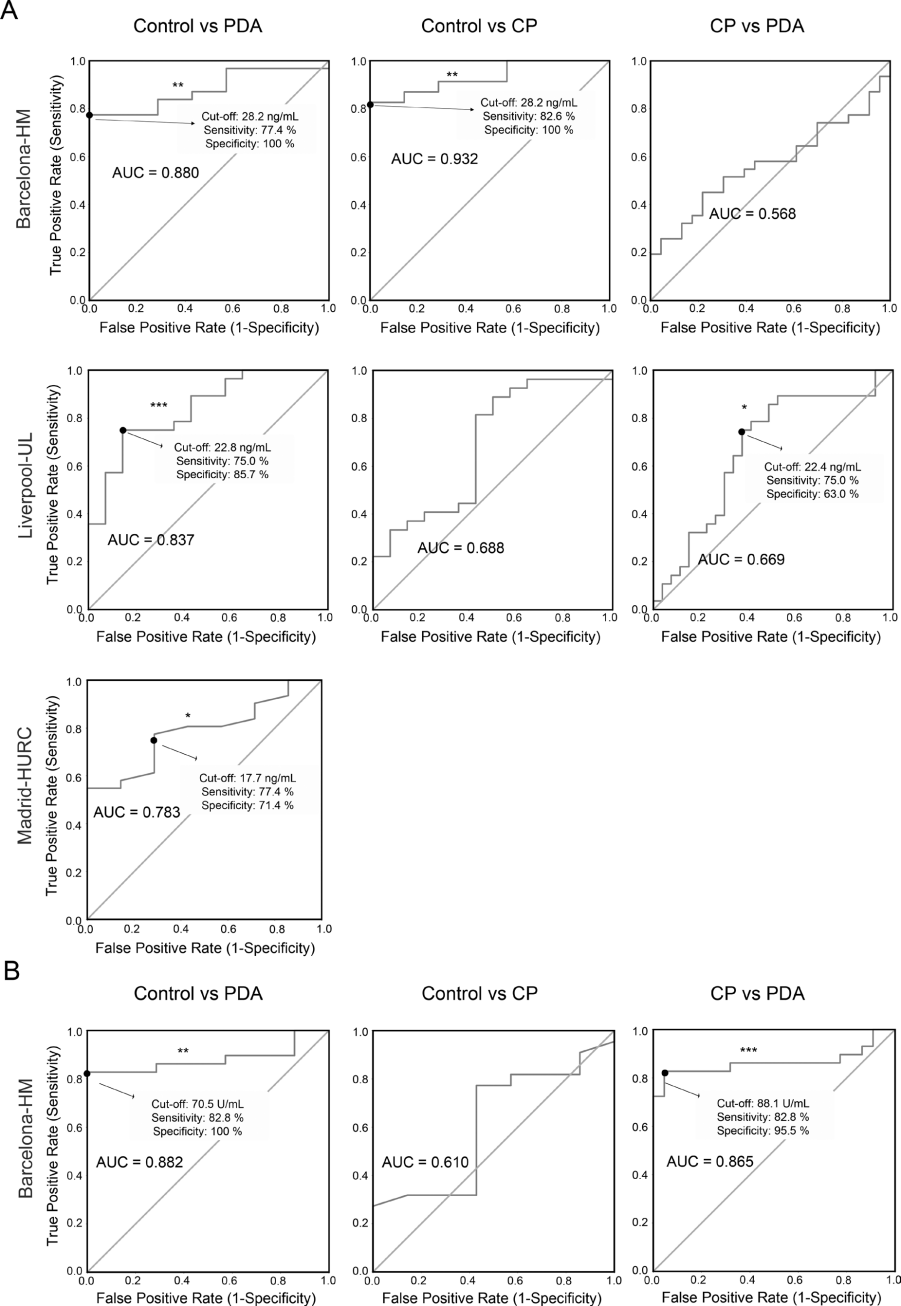
ROC curves for determining specificity and sensitivity values for Gal-1 and CA19-9 (**A**) ROC curves for Gal1 in the three cohorts. (**B**) ROC curves for CA19-9 in the Barcelona-HM cohort.

Receiver operating characteristic (ROC) curves were used to: i) calculate the usefulness of Gal-1 as a diagnostic marker (with area under curve [AUC]); and ii) determine the optimal cut-off value of Gal-1 levels in plasma for PDA detection. In the Barcelona-HM cohort, significant ROC curves with AUC values of 0.932 and 0.880 were derived from control (Ctl) and CP/PDA data, respectively (Figure 3A and Supplementary Table 6). We found similar cut-off values (maximizing Younden index) [41] of 28.16 ng/ml (82.6% sensitivity and 100% specificity) for the Ctl-CP comparison, and of 28.15 ng/ml (77.4% sensitivity and 100% specificity) for the Ctl-PDA comparison (Figure 3A and Supplementary Table 6). In the Liverpool-UL cohort, significant ROC curves were obtained from Ctl-PDA data (AUC, 0.837; cut-off 22.83 ng/ml, with 75% sensitivity and 85.7% specificity) and from CP-PDA data (AUC, 0.669; cut-off 22.4 ng/ml, with 75% sensitivity and 63% specificity) (Figure 3A and Supplementary Table 6). Due to the low number of CP patients in the Madrid-HURC cohort, ROC curves were only used to compare values between PDA patients and healthy individuals; this showed an AUC of 0.783 with a maximum Youden index at 21.3 ng/ml (54.8% sensitivity and 100% specificity). However, a cut-off value of 17.7 ng/ml balanced sensitivity and specificity (at 77.4% and 71.4%, respectively) (Figure 3A and Supplementary Table 6).

### Levels of CA19-9 and Gal-1 in plasma from healthy individuals as compared to patients with CP or PDA

Detection by ELISA of CA19-9 antigen in blood is the only blood biomarker approved by the FDA for PDA diagnosis. Notably, CA19-9 has a poor-to-moderate sensitivity (70%–80%) and specificity (68%–91%) for PDA, and it is only recommended for monitoring patient therapy response but not for primary diagnostics [10]. To investigate whether detection of plasma Gal-1 is more sensitive than that of CA19-9, we measured the plasma levels of CA19-9 in healthy control individuals and patients with CP or PDA from Barcelona-HM. CA19-9 antigen was significantly elevated in PDA patients (405.1 U/ml) as compared to healthy controls (9.0 U/ml) or CP patients (13.3 U/ml) (Supplementary Table 7). We next compared sensitivity and specificity of CA19-9 versus Gal-1 levels in plasma for CP and PDA detection using ROC curves. Samples from control individuals or patients with CP or PDA from Barcelona-HM were used to generate ROC curves and to determine the optimal cut-off value of CA19-9 for PDA detection. CA19-9 serum levels of 70.5 U/ml showed 82.8% sensitivity and 100% specificity for PDA samples (Figure 3B and Supplementary Table 6). Interestingly, AUC sensitivity and specificity values for both Gal-1 and CA19-9 markers were comparable, and the number of false negatives was greatly reduced when both biomarkers were used together, with an increase up to 96% sensitivity and 100% specificity. Gal-1, but not CA19-9, was able to identify CP patients from the healthy population in the Barcelona-HM cohort (Figure 3 and Supplementary Table 6).

These data indicate that measuring Gal-1 levels in patient plasma presents a novel independent biomarker for PDA detection, and that Gal-1 could be used as a complementary blood marker in PDA diagnosis; notably, combining Gal-1 and CA19-9 detection could drastically decrease cases of false-negative diagnoses of PDA after an initial CA19-9 test.

### Plasma Gal-1 levels during PDA progression and for prognosis

**Figure 4:**
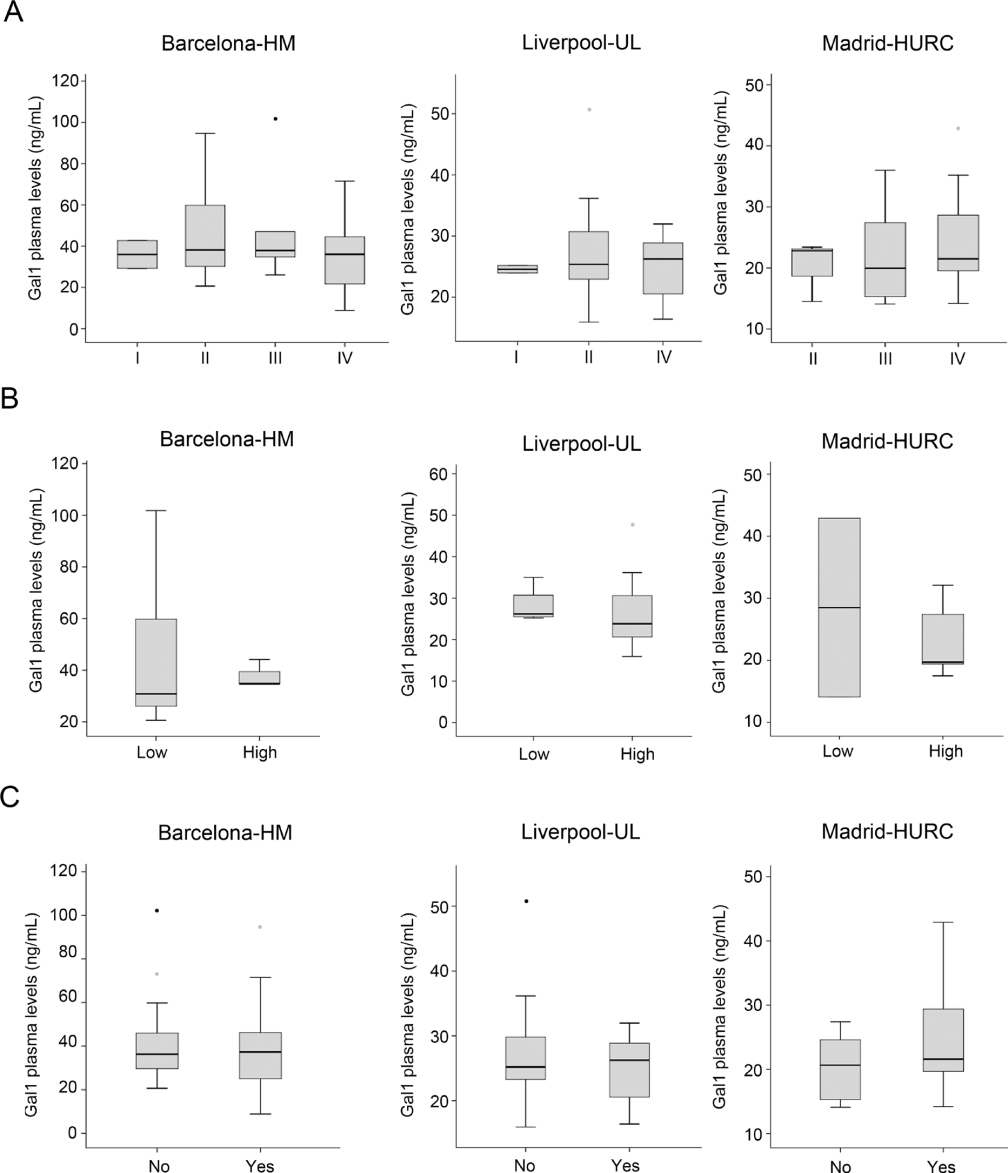
Comparison of the plasma Gal-1 leves with respect to tumor stage, tumor grade and metastasis (**A**) PDA patients segregated by TNM tumor stage and their respective Gal-1 plasma levels, as shown by box-and-whisker plots. (**B**) PDA patients segregated by tumor grade (low or high) and their respective Gal-1 plasma levels, as shown by box-and-whisker plots. (**C**) Gal-1 plasma levels in patients without (“no”) or with (“yes”) metastasis.

To explore whether circulating Gal-1 levels is predictive for PDA tumor progression, PDA patients were stratified by tumor stage following TNM classification [42], and Gal-1 plasma concentrations were compared between groups. No significant correlations were found between circulating Gal-1 and tumor stage (Figure 4A). Similarly, no differences between subpopulations were observed in Gal-1 levels when patients were stratified by tumor grade (Figure 4B) or presence of metastasis (Figure 4C).

**Figure 5:**
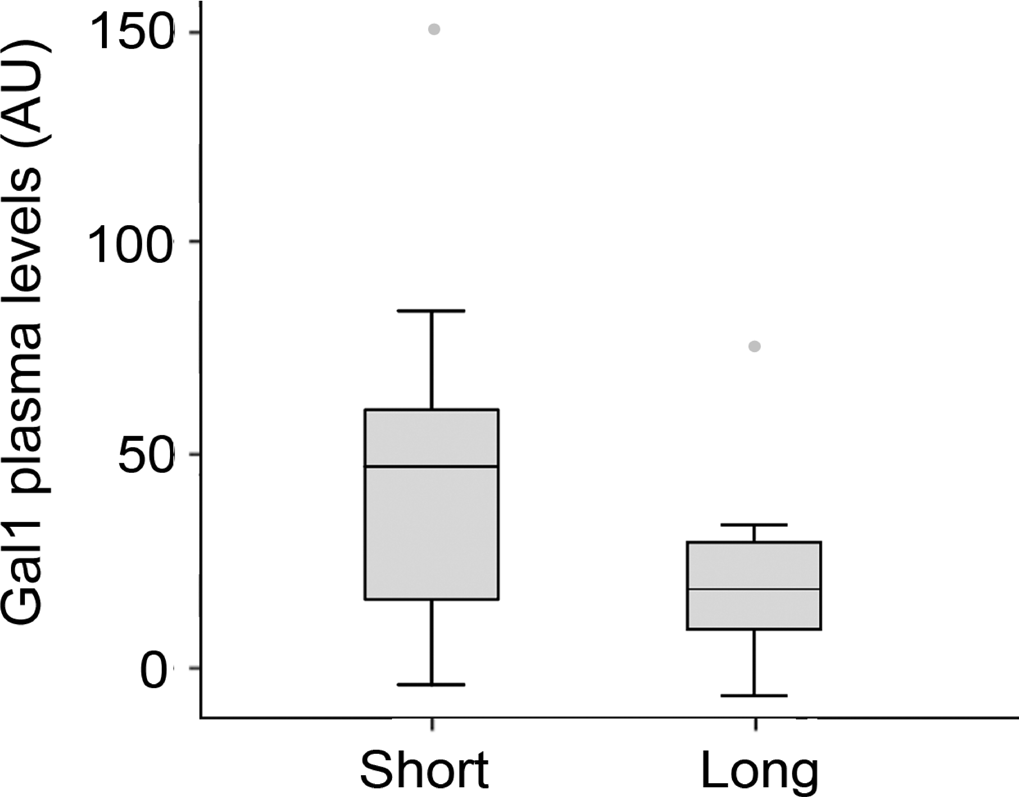
Gal-1 plasma levels in PDA patients displaying short-(<6 months) or long-term survival (≥6 months)

Next, we evaluated whether Gal-1 levels correlate with PDA prognosis and patient overall survival. Patients with unresectable tumors were classified as short-term (<6 months) or long-term (≥6 months) survivors, and plasma Gal-1 levels were determined for each group. Due to low sample number, Gal-1 levels were standardized in the three cohorts to allow comparison of Gal-1 ELISA results. Although no statistical significance was reached, a trend was observed for increased Gal-1 levels in short-term survivors as compared to long-term survivors (Figure 5 and Supplementary Table 8). Thus, testing for plasma Gal-1 levels may have prognostic value for PDA patients with unresectable tumors.

## DISCUSSION

PDA is one of the most aggressive tumors, with a 5-year survival rate of less than 8% [43]. In contrast to other tumors, this cancer has not benefited from any significant clinical advances in the last years, and more than 80% of patients are diagnosed at advanced stages, at which point surgical resection (the only potential curative treatment) is not possible. Among the different strategies for tumor detection, serum biomarkers offer many advantages for inclusion in routine analysis, such as ease of sample collection, minimal invasiveness for patients and low cost. Currently, the CA19-9 antigen is the only blood-based biomarker approved for PDA, although its use is only recommended for monitoring patient therapy response [10]. Two major concerns have been raised about the use of CA19-9 for PDA diagnosis: i) elevated CA19-9 levels in blood are found for obstructive biliary diseases, inflammatory processes and other tumors [9, 10, 44], leading to only moderate sensitivity (70%–80%) and specificity (68%–91%) for PDA; and ii) CA19-9 cannot be used for the Lewis^a–^ population, who represent 5%–10% of the Caucasian population [8, 45]. These drawbacks strongly limit the effectiveness of CA19-9 levels as a PDA biomarker due to high rates of false positive results as well as (for the Lewis^a–^ population) false negative results.

Gal-1 is a small soluble molecule that is rapidly secreted to the extracellular space by a non-canonical secretory pathway [29], suggesting that high protein levels observed in tissue samples from patients with pancreatitis and PDA would also be found in their blood. We demonstrate here that ELISA detected plasma Gal-1 levels that mirrored Gal-1 tissue expression levels, making Gal-1 a good biomarker for PDA. While Gal-1 displayed similar sensitivity and specificity values as CA19-9, the combination of both biomarkers strongly reduced the number of diagnosed false negatives in our study. Moreover, ROC analysis to discriminate PDA from pancreatitis was also significantly improved using Gal-1 ELISA measurements as compared to using only CA19-9. Further, we observed that PDA short-term survivors were more likely to have higher Gal-1 plasma levels, suggesting that this biomarker could have a prognostic value. Altogether, our study identified the detection of circulating Gal-1 as a novel biomarker for pancreatic cancer with putative translational applications for diagnosis and prognosis of this fatal disease.

Our exhaustive IHC analysis of the expression levels of Gal-1 in normal pancreas, CP, preneoplastic lesions (IPMN and PanIN) and PDA samples showed similar high Gal-1 levels that were mostly restricted to fibrotic stroma in all pathological situations. These results contrast with previous studies that detected increased Gal-1 expression in PDA tissue samples as compared to pancreatitis by IHC [46] and by quantitative proteomics and mass spectrometry [22]. These discrepancies could be attributed to technical reasons, differences in patient tissue samples or different quantification scores used in each study. Of note, our quantification of Gal-1 expression was restricted to pathological lesions (i.e., the surrounding normal tissue was discarded) and was calculated by multiplying H-scores and stroma percentage. Increased Gal-1 levels in pancreatitis and PDA might reflect the higher proportion of fibrosis in these diseases as compared to preneoplastic lesions (PanINs and IPMNs).

Importantly, Gal-1 expression levels in tissue samples are at least partially mirrored by its levels in blood. Gal-1 levels detected by ELISA in plasma from patients with PDA were significantly increased as compared to healthy individuals (who had values of around 18 ng/ml, consistent with previous studies [47]). However, we did not find changes associated with TNM or differentiation grade of PDA tumors, indicating that plasma Gal-1 levels might reflect tumor burden (and associated fibrosis) rather than cancer progression. These results are in agreement with previous data showing that circulating Gal-1 levels were elevated at early stages of colorectal cancer as compared to normal tissue but did not significantly change during tumor progression [33]. Interestingly, Gal-1 expression in colorectal cancer is also found in cancer-associated fibroblasts, suggesting a similar scenario to pancreatic tumors. Notably, blood levels of Gal-1 in patients with CP were higher than those for healthy controls, but lower than those for patients with PDA (note that the significance of these findings could only be verified in Barcelona-HM or Liverpool-UL, respectively, due to low patient numbers; see Figure 3). The apparent contrast of these results and those from our tissue samples tested by IHC (in which both CP and PDA had similar Gal-1 expression levels) is likely due to the differences in severity of disease for patients analyzed by the two methods: tissue samples were obtained after surgical interventions of severe CP and showed extensive fibrotic areas, while blood samples were obtained from patients diagnosed with CP by echo-endoscopy, who are less likely to require surgery and present less inflammatory areas (and, consequently, have lower levels of Gal-1). Discriminating between CP and PDA is one of the most important challenges for gastroenterologists. PDA is frequently asymptomatic at early stages but, after tumor progression, patients report symptoms similar to those for CP, such that accurate diagnosis requires imaging or molecular techniques. Although limited by the small sample number in the cohorts, our data suggest that measuring Gal-1 plasma levels can discriminate between patients with CP and those with PDA. Future studies with larger number of patients are required to confirm the suitability of Gal-1 as a biomarker for distinguishing PDA from CP. Intriguingly, however, CP has a strong link with the development of PDA, and around 5% of patients with CP develop pancreatic cancer [48], making it tempting to speculate that plasma Gal-1 detection would be a valuable tool for screening patients with CP to identify risk of possible tumor development.

A major function of Gal-1 is modulating the immune response, both in physiological and pathological conditions. In particular, Gal-1 has anti-inflammatory and immunosuppressive effects [17], suppresses recruitment and extravasation of neutrophils, induces the macrophage switch from M1 to M2 phenotype, promotes tolerogenic dendritic cells and induces T-regulatory cell differentiation [49, 50]. These roles of Gal-1 are determinant for PDA progression [25, 51, 52]. Our immunohistochemical analysis now indicates that Gal-1 expression is an early event in pancreatic pathologies associated to stroma activation, such as inflammation and preneoplasia. Considering the critical role of Gal-1 in driving immune evasion, we speculate that expression of this lectin in the activated stroma associated to pancreatitis or preneoplastic lesions can promote immune privilege, which hampers the immune system from recognizing initiating tumor cells and thereby promotes immune escape, tumor onset and progression. This hypothesis might also explain the increased risk of PDA in patients with CP. Moreover, the potential of the lectin as a novel target in PDA cannot be underestimated, considering that PDA development in a Gal-1 knockout background is significantly delayed [24, 25], and that its inhibition (using peptides, glycan-based inhibitors or more specific monoclonal antibodies) has a proven efficacy for other tumors [19, 53–60].

Increased levels of circulating Gal-1 have been reported in several other tumors [30–35]. However, Gal-1 is expressed by tumor epithelial cells in most of these cases, while it is expressed mostly by stromal pancreatic stellate cells in PDA. These data indicate that stellate cells/fibroblasts play a relevant role for PDA biology, and that proteins expressed by non-epithelial cells could also represent useful biomarkers for cancer, which would expand the number of proteins to be considered as candidates for tumor diagnosis. In fact, most of the previously reported biomarkers for PDA (e.g., CA19-9, CEA, α-fetoprotein, MMP-7, cathepsin D, integrin B1, HSP27, elastase-1, MCSF and CA195) are expressed by epithelial tumor cells, while Gal-1 is specifically expressed and released by pancreatic-activated fibroblasts. Mounting evidence indicates that a “unique” biomarker for a specific cancer does not exist; for PDA, a panel of biomarkers would likely be a more reliable way of overcoming the limited specificity and sensitivity of the PDA biomarkers identified to date. Indeed, we found that co-analysis of CA19-9 and Gal-1 increased sensitivity as compared to using either biomarker separately.

Although diabetes is frequent in PDA patients, we did not observe any correlation between diabetes and increased Gal-1 levels (Supplementary Table 5), despite reports of increased Gal-1 levels in type II diabetes patients [40]. Increased levels of CA19-9 have been also reported in up to 50% of diabetic patients [61] and in PDA patients with diabetes [62], suggesting additional advantages of using Gal-1 as a PDA biomarker in conjunction with CA19-9 rather than CA19-9 alone.

In conclusion, our study demonstrates that measurement of Gal-1 levels by ELISA is a novel method for improving PDA diagnosis, which could eventually lead to predicting prognosis of patients with unresectable tumors. In addition, high Gal-1 levels in blood from PDA patients suggest that it could be a useful marker for patient follow-up—e.g., for detecting recurrence after surgery and for evaluating tumor response during chemotherapy or chemo/radiotherapy, as reported for other tumor types [33, 34]. ELISA is easy-to-use and cost effective, and blood collection is minimally invasive, underscoring the strong potential of these results. Future research using multicenter trials with large patient numbers is now necessary to establish the clinical impact of using circulating Gal-1 levels as novel biomarker for PDA diagnosis and follow-up.

## MATERIALS AND METHODS

### Patients, tissue samples, and blood plasma collection

For histological studies, samples from normal pancreas (*n* = 19), CP (*n* = 13), preneoplastic lesions (IPMNs, *n* = 7 and PanINs, *n* = 9), and PDA (*n* = 30) were obtained from Parc de Salut MAR Biobank (MARBiobanc), Barcelona; clinicopathological characteristics of these tissue samples are summarized in the Supplementary Table 1. Blood samples were collected from three different cohorts: Barcelona-Hospital del Mar (HM) cohort (comprising 7 controls, 23 patients with CP, and 31 patients with PDA), Liverpool-University of Liverpool (UL) cohort (comprising 14 controls, 27 patients with CP, and 28 patients with PDA), and Madrid-Hospital Universitario Ramón y Cajal (HURC) cohort (comprising 7 controls, 2 patients with CP, and 31 patients with PDA); the clinical data corresponding to the plasma samples included in this study are summarized (Supplementary Table 2). This study was evaluated and approved by the Clinical Research Ethical Committee of the Parc de Salut Mar (CEIC-Parc de Salut Mar), Health Research Authority (Liverpool), and Ramon y Cajal CEIC, Madrid. All individual participants in the study voluntarily signed an informed consent allowing the use of their samples for research.

### Immunohistochemistry (IHC)

Paraffin sections (3 μm) were used for IHC analysis, as described [25]. Rabbit α-Gal-1 polyclonal antibody (Abcam) or irrelevant IgG (as negative control) were used as primary antibodies, and HRP-anti-rabbit-EnVision (DAKO, EnVision™+ System) as a secondary antibody. Immunostainings were analyzed by two experts in pancreatic pathology, who recorded intensity and percentage of stained cells to calculate the H-scores [63]. The percentage of stroma in each sample was quantified using Image J software analysis, and its value was used to normalize the H-score.

### Measurement of plasma levels of Gal-1 and CA19-9 by ELISA

Plasma Gal-1 levels of were quantified with the human Gal-1 ELISA kit (R&D systems) according to manufacturers' protocols. Plasma samples were diluted 1:10 for the analysis. Gal-1 was detected by absorbance determination at 450–570 nm using an ELISA reader (200 series, Tecan). Serum levels of CA19-9 were measured by ELISA kit (Cobas) at the Laboratori de Referència de Catalunya (Barcelona, Spain).

### Statistical analysis

Data analyses were carried out using the SPSS software (IBM SPSS statistics version 23). Statistical significance was set at *p* < 0.05 (^*^p < 0.05; ^**^p < 0.01; ^***^p < 0.001). As ELISA measurements of Gal-1 and CA19-9 showed skewed distributions, data are described as median and interquartile range (IQR), and the nonparametric analyses of Mann-Whitney were applied. For bivariate correlations, the Spearman test was used. Receiver operating characteristic (ROC) curve analysis was used to determine the cut-off values to detect the predictive power of Gal-1 or CA19-9, in order to discriminate PDA from CP or healthy controls. Results are given as area under curve (AUC) with 95% confidence limits. For survival analysis, data in each cohort were standardized using:

$$x=\frac{X-\text{Mean}\left(\text{Ctl}\right)}{\text{SD}\left(\text{Ctl}\right)}\cdot 100$$

## SUPPLEMENTARY MATERIALS TABLES



## Abbreviations

CP: chronic pancreatitis
HM: Hospital del Mar
HURC: Hospital Universitario Ramón y Cajal
IHC: immunohistochemistrys
IPMN: intraductal papillary mucinous neoplasm
PDA: pancreatic ductal adenocarcinoma
PanINs: pancreatic intraepithelial neoplasia
UL: University of Liverpool
