# Supplementary material for: Increased plasma levels of galectin-1 in pancreatic cancer: potential use as biomarker
Source: Oncotarget. 2018 Aug 31;9(68):32984–96. doi: 10.18632/oncotarget.26034 (PMC6152472; doi:10.18632/oncotarget.26034)
Supplement: Supplementary file 1 [file oncotarget-09-32984-s001.pdf]

Increased plasma levels of galectin-1 in pancreatic cancer: potential use as biomarker

SUPPLEMENTARY MATERIALS

Supplementary Table 1: Summary of the clinicopathological data of patients included in the analysis of the quantification of Gal-1 expression by IHC in tissue

		n	Median	IQR	*p*
**Age**					
	**≤70**	30	41.80	42.92	
	**>70**	48	32.23	41.73	ns
**Sex**					
	**Male**	45	33.70	50.04	
	**Female**	33	37.75	40.38	ns
**Pathology**					
	**Healthy**	19	4.60	6.06	
	**Pancreatitis**	13	59.33	27.07	<0.001
	**PanIN**	9	29.77	14.82	<0.001
	**IPMN**	7	25.76	16.14	<0.001
	**PDA**	30	54.29	26.61	<0.001
**TNM**					
	**0**	1	63.46	-	
	**1A**	2	68.75	-	ns
	**2A**	7	58.52	21.72	ns
	**2B**	16	54.29	18.53	ns
	**3**	3	32.60	-	ns/0.023*
	**4**	1	31.76	-	ns
**Grade**					
	**1**	5	61.47	41.94	
	**2**	12	49.00	35.85	ns
	**3**	13	55.86	19.43	ns
**Survival**					
	**<2 years**	12	54.28	28.78	
	**≥2 years**	16	50.66	29.52	ns

p, comparison with respect to the first category of the group being evaluated; **p*, comparison of TNM 2B to TNM 3; ns, not significant.

Supplementary Table 2: Clinicopathological data of patients included for Gal-1 detection in plasma samples by ELISA

		Barcelona-HM			Liverpool-UL			Madrid-HUMC		
		n	Sex (M/F)	Age (Range)	*n*	Sex (M/F)	Age (Range)	*n*	Sex (M/F)	Age (Range)
**Pathology**										
**Control**		7	(4/3)	(38–75)	14	(9/5)	(49–66)	7	(4/3)	(48–55)
**CP**		23	(17/6)	(27–84)	27	(18/9)	(26–76)	2	(0/2)	(50–56)
**PDA**		31	(17/14)	(51–90)	28	(15/13)	(42–88)	31	(15/16)	(42–90)
**Stage**										
	**I**	2			2			0		
	**II**	10			18			3		
	**III**	5			0			6		
	**IV**	14			8			20		
	**NA**	0			0			2		
**Grade**										
	**Low**	10			5			2		
	**High**	3			18			9		
	**N/A**	18			5			20		
**Metastasis**										
	**No**	17			20			10		
	**Yes**	14			8			21		

N/A: not ascertained.

Supplementary Table 3: Analysis of Gal-1 plasma levels according to age in the healthy controls of the three cohorts

Cohort	*n*	Mean	SD	Min	Max	*p* (Spearman)
**Barcelona-HM**	7	56.43	11.30	38	75	0.819
**Liverpool-UL**	14	56.86	4.94	49	66	0.265
**Madrid-HURC**	7	51.57	3.21	48	55	0.857

$p > 0.05$ in all cases; Spearman correlation.

Supplementary Table 4: Comparison of Gal-1 plasma levels between men and women in the healthy controls of the three cohorts

Cohort	Sex	*n*	Median	IQR	*p* (Mann–Whitney)
**Barcelona-HM**	Male	4	13.02	14.11	
	Female	3	23.25	-	0.4
**Liverpool-UL**	Male	9	17.47	7.47	
	Female	5	13.39	9.31	0.112
**Madrid-HUMC**	Male	4	16.00	7.72	
	Female	3	16.00	-	0.857

$p > 0.05$ in all cases; Mann–Whitney test.

Supplementary Table 5: Gal-1 plasma levels according to bilirubin levels or diabetes status in PDA patients of the three cohorts

	Barcelona-HM				Liverpool-UL				Madrid-HUMC			
	n	Median	IQR	*p*	*n*	Median	IQR	*p*	*n*	Median	IQR	*p*
Bilirubin												
**Normal**	23	34.74	15.64		23	23.91	12.88		22	20.85	9.0	
**High**	9	44.14	26.65	ns	28	28.832	7.44	ns	9	23.4	18.0	ns
Diabetes												
**No**	21	30.29	12.31		49	21.42	9.58		23	21.6	13.0	
**yes**	15	42.68	19.56	ns	20	24.865	10.97	ns	10	19.9	5.0	ns

ns, not significant.

Supplementary Table 6: Data for ROC analysis for Gal-1 (three cohorts) and CA19-9 (Barcelona-HM cohort)

Cohort		Groups	AUC	SE	*p*	Cut-off	Sensitivity	Specificity
**Barcelona-HM**	**Gal-1**							
		Ctl vs CP	0.932	0.045	0.001	28.16	82.6	100
		Ctl vs PDA	0.880	0.056	0.002	28.15	77.4	100
		CP vs PDA	0.568	0.078	0.396	-	-	-
**Barcelona-HM**	**CA19-9**							
		Ctl vs CP	0.610	0.122	0.386	-	-	-
		Ctl vs PDA	0.882	0.056	0.002	70.5	82.8	100
		CP vs PDA	0.865	0.057	<0.0001	88.1	82.8	95.5
**Liverpool-UL**	**Gal-1**							
		Ctl vs CP	0.688	0.091	0.051	16.86	88.9	50
		Ctl vs PDA	0.837	0.064	<0.0001	22.83	75	85.7
		CP vs PDA	0.669	0.075	0.031	22.39	75	63
**Madrid-HURC**	**Gal-1**							
		Ctl vs PDA	0.783	0.079	0.021	21.3	54.8	100

AUC, area under curve; CP, chronic pancreatitis group; Ctl, control group; PDA, pancreatic ductal adenocarcinoma group; SE, standard error associated with AUC.

Supplementary Table 7: CA19-9 levels in Barcelona-HM cohort

	n	Median	IQR	*p*
**Control**	7	9.00	22.40	
**CP**	22	13.30	28.05	0.409
**PDA**	29	405.10	689.95	0.001/0.000*

*p value of CP compared to PDA. CP, chronic pancreatitis group; PDA, pancreatic ductal adenocarcinoma group.

Supplementary Table 8: Correlation of Gal-1 plasma levels (standardized in three cohorts) and survival of PDA patients

Gal-1 standardized	*n*	Median	IQR	*p*
**Short term (<6 months)**	16	4.67	5.47	
**Long term (≥6 months)**	14	1.80	2.02	0.09
